# Systematic Pharmacology Combined With Experimental Validation Reveals the Apoptosis‐Related Mechanism of Simiao Yong′an (SMYA) Decoction Against Myocardial Ischemia‐Reperfusion Injury

**DOI:** 10.1155/cdr/4200440

**Published:** 2026-05-05

**Authors:** Huanjie Fu, Eryue Liu, Hao Yu, Yisheng Zhao, Yongkang Gan, Jinhong Chen, Zhichao Liu

**Affiliations:** ^1^ Department of Cardiovascular, Second Affiliated Hospital of Tianjin University of Traditional Chinese Medicine, Tianjin, China, tjutcm.edu.cn; ^2^ Department of Pharmacy, First Teaching Hospital of Tianjin University of Traditional Chinese Medicine, Tianjin, China, tjtcm.cn; ^3^ National Clinical Research Center for Chinese Medicine, Acupuncture and Moxbustion, Tianjin, China, >; ^4^ Intensive Care Unit, Second Affiliated Hospital of Tianjin University of Traditional Chinese Medicine, Tianjin, China, tjutcm.edu.cn; ^5^ Department of Vascular Surgery, Tianjin Academy of Traditional Chinese Medicine Affiliated Hospital, Tianjin, China; ^6^ School of Rehabilitation Medicine, Shandong Second Medical University, Weifang, Shandong Province, China

**Keywords:** acute myocardial infarction, PI3K-AKT, Simiao Yong′an decoction, therapeutic targets of active compounds

## Abstract

**Background:**

According to traditional Chinese medicine theory, acute myocardial infarction (AMI) is primarily associated with qi stagnation and blood stasis. Simiao Yong′an (SMYA) decoction is a well‐known prescription that clears heat, detoxifies, and promotes blood circulation. SMYA has been used in the treatment of ischemic heart diseases (IHD). However, further analysis is required to clarify the specific mechanisms through which SMYA improves AMI and to determine its therapeutic effects at different time points during the acute phase of myocardial infarction.

**Aim of This Study:**

This study is aimed at investigating the protective effects of SMYA against AMI at various time points and to explore its underlying mechanisms.

**Materials and Methods:**

The active ingredients in SMYA were identified through ultraperformance liquid chromatography‐quadrupole‐time‐of‐flight mass spectrometry (UPLC‐Q‐TOF/MS). An integrated in silico approach was employed to predict potential targets of these compounds, and target‐pathway associations were established by aligning the data with relevant databases. A cardiac ischemia/reperfusion (I/R) model in rats was created by ligating the left coronary artery, inducing ischemia for 45 min, and allowing for 24 h of reperfusion. SMYA treatment was administered for 7 days. Cardiac function was evaluated at different time points during the acute phase of myocardial infarction using echocardiography. Serum biochemical indexes were measured using a biochemical kit, and western blotting (WB) was used to analyze AKT, p‐AKT, PI3K, p‐PI3K, BAX, Bcl‐2, and caspase‐3 proteins.

**Results:**

UPLC‐Q‐TOF/MS identified 25 components in SMYA, which were considered potential effective ingredients. Network analysis identified 161 key targets and 167 Kyoto Encyclopedia of Genes and Genomes (KEGG) pathways associated with SMYA, with the PI3K‐Akt pathway being notably prominent. Experimental validation demonstrated that SMYA significantly reduced the levels of creatine kinase isoenzyme (CK‐MB) and lactate dehydrogenase (LDH) in serum and improved left ventricular ejection fraction (LVEF) and fractional shortening (FS) after myocardial I/R injury in rats. Additionally, SMYA reduced myocardial cell apoptosis and activated the PI3K‐AKT pathway in a dose‐dependent manner. Molecular docking confirmed binding between SMYA components and AKT/BCL‐2.

**Conclusion:**

This study elucidates the mechanisms underlying AMI and the molecular action of SMYA. SMYA alleviates I/R‐induced AMI in rats by activating the PI3K‐AKT pathway, suggesting its potential as a therapeutic target for myocardial remodeling. The dose‐ and time‐dependent protective effects of SMYA suggest that the PI3K‐AKT pathway and its downstream target BCL‐2 constitute promising therapeutic targets for novel interventions in AMI.

## 1. Introduction

From a pathological perspective, acute coronary syndromes (ACS) are defined by a sudden reduction in blood supply to the heart, encompassing ST‐segment elevation myocardial infarction (STEMI), non–ST‐segment elevation myocardial infarction (NSTEMI), and unstable angina. Globally, more than 7 million individuals are diagnosed with ACS each year [1]. Although cardiovascular mortality post‐myocardial infarction (MI) significantly decreased between 2000 and 2017, the risk of noncardiovascular morbidity has notably increased [2]. Noncardiovascular factors play a substantial role in post‐MI mortality, underscoring the need for greater attention to noncardiovascular outcomes in both clinical practice and the design of future clinical guidelines [3].

In recent decades, advancements in pharmacological treatments, catheter‐based interventions, and surgical reperfusion have significantly improved outcomes for patients with acute myocardial infarction (AMI) [4]. However, immediate reperfusion can paradoxically exacerbate myocardial injury, resulting in ischemia/reperfusion (I/R) injury [5]. Reperfusion injury may lead to further myocardial damage, including reperfusion arrhythmias, no‐reflow phenomena, and severe myocardial tissue injury that exceeds that caused by the initial ischemic event [6]. Although PCI does not represent the final step in MI treatment, it remains a cornerstone despite its associated risks [7, 8]. This is especially true for patients with large infarcts or those who do not receive early revascularization, who are at heightened risk of mortality [4]. Consequently, there is substantial interest in developing effective strategies to improve the efficacy and prognosis of MI treatments. Complementary and alternative therapies, particularly those derived from traditional Chinese medicine (TCM), have gained increasing attention as promising sources for innovative treatments. As a cornerstone of Chinese healthcare, TCM is gaining recognition within modern medicine for its potential in drug discovery [9, 10]. However, challenges such as unclear drug composition and mechanisms of action have impeded its broader clinical adoption, limiting potential clinical advancements [11].

TCM has a long history of use in the treatment of cardiovascular diseases, with numerous herbal formulations demonstrating significant therapeutic potential. Simiao Yong′an (SMYA) decoction, a well‐known TCM formula, consists of honeysuckle, radix scrophulariae, *Angelica sinensis*, and liquorice (Table 1). This decoction has traditionally been used to clear heat, detoxify, and promote blood circulation [15–17]. The effects of SMYA decoction on coronary heart disease (CHD) are well‐established and widely studied, particularly in relation to anti‐inflammatory effects [12, 13, 18, 19]. Recent studies have revealed the cardiomyocyte‐protective effects of SMYA through activation of autophagy and inhibition of NLRP3‐related pyroptosis [20]. Relevant studies using network pharmacology and transcriptome analyses have predicted and demonstrated some of the mechanisms underlying the anti‐I/R effects of SMYA. Previous research has indicated that SMYA possesses anti‐inflammatory, antioxidant, and cardioprotective properties, suggesting its potential for treating myocardial I/R injury [21, 22]. SMYA has been found to inhibit the LPS‐induced TLR4/NF‐*κ*B pathway, thereby reducing the release of inflammatory factors and alleviating myocardial injury [23]. In vitro studies further suggest that quercetin confers protective effects in a cardiomyocyte injury model, potentially through the upregulation of phosphorylated AKT1 expression [24]. Additional evidence indicates that SMYA can inhibit oxidative stress, improve cardiac injury, and reduce the release of NLRP3‐related inflammatory cytokines [25]. Nevertheless, the specific active components and detailed mechanisms responsible for its therapeutic effects remain largely unelucidated.

**Table 1:** Detailed information of herbs in SMYA (for human).

Chinese name	Latin name	Part(s) used	Ratio
Jinyinhua	*Lonicera japonica* Thunb.	Buds and newly blooming flowers	3
Xuanshen	*Scrophularia ningpoensis* Hemsl.	Roots	3
Danggui	*Angelica sinensis* (Oliv.) Diels	Roots	2
Gancao	*Glycyrrhiza* uralensis Fisch.	Roots and rhizomes	1

*Note:* The dosage ratio of the medication was based on prior studies [12–14].

**Figure 1:**
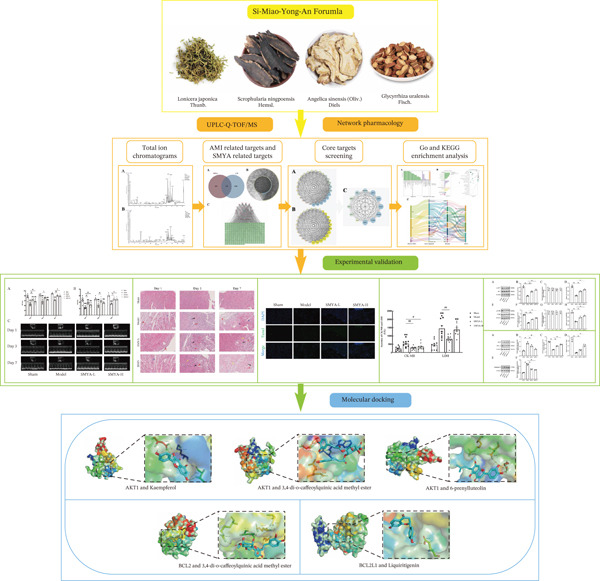
Flowchart of this study.

To further elucidate the cardioprotective mechanisms of SMYA, we evaluated its effects on cardiac function using a rat model of cardiac I/R injury. A comprehensive pharmacological approach was used to investigate the mechanisms of the therapeutic action of SMYA against I/R injury. Potential bioactive components of SMYA were analyzed using the UPLC‐Q‐TOF/MS technique and a compound‐target network was established with the help of topology analysis to predict possible targets. These targets were subsequently analyzed using relevant databases to unveil associated pathways. Bioinformatics and molecular biology methods have been used to perform the required validation assays for these predictions. The results obtained using integrated approaches provided initial valuable insights into the potential clinical applications of SMYA and its constituents in relation to their proposed therapeutic roles in the acute phase of I/R injury. In this review, we will update the knowledge on the cardioprotective effects of SMYA decoction, its possible mechanisms, and its potential use as an alternative or complementary therapy for myocardial I/R injury (Figure 1). The findings from this study may lay the groundwork for novel therapeutic strategies to improve cardiac outcomes in patients with ischemic heart disease.

## 2. Materials and Methods

### 2.1. Animals and Preparation of Plasma Samples

The Sprague–Dawley rat model for cardiac I/R injury was established using rats weighing 250–300g. Every rat was kept in a clean, germ‐free environment with a temperature of 22^°^C ± 2^°^C, relative humidity of 50%–70%, and a 12‐h light/dark cycle. Tianjin Jinke Bona Biotechnology Co. LTD.′s Experimental Animal Ethics Committee gave its stamp of approval to the study′s procedures (Approval No. GENINK20230060). The Procedures for the Humane Treatment of Laboratory Animals were adhered to in all investigations. Rats were randomly allocated to the sham, model, SMYA‐L, and SMYA‐H groups. Sample sizes were chosen based on previous similar studies [10, 26]. The 36 rats were divided into four groups: sham (*n* = 9), model (*n* = 9), low‐dose (*n* = 9) and high‐dose (*n* = 9) SMYA‐treated (SMYA‐L and SMYA‐H) groups after acclimatization took place for 7 days. Ischemia for 45 min and subsequent reperfusion for 24 h caused cardiac I/R injury. Just to recap, male rats were given a 2% avertin injection (0.1 mL/10 g) intraperitoneally (i.p.) to put them to sleep. The rats that were put to sleep were intubated and given oxygen using a rodent ventilator (R415; RWD Life Science, China) that was adjusted to 110 breaths per minute and a tidal volume of 200 *μ*L. Thoracotomy was performed to expose the heart through the third and fourth intercostal gaps after disinfecting the left chest skin. A 7–0 silk suture was used to tie a slipknot 1–2 mm from the lower margin of the left atrium, occluding the left anterior descending coronary artery (LAD). Release of the slipknot allowed for 24 h of reperfusion after 45 min [27]. The sham group underwent the identical process but did not undergo ligation. A heated blanket was used to keep the animals warm after the operation.

The four constituents of SMYA infusion included honeysuckle, *A. sinensis*, licorice, and radix scrophulariae. The total time of boiling in a pot of pure water with the herbs was 2 and 1 h, respectively. The *Lonicera japonica*, radix scrophulariae, *A. sinensis*, and liquorice were distilled together in a ratio of 3:3:2:1, respectively, and then condensed to 2 g/mL. We used exactly the same methodology for our preparation as that described by Zhao et al. [14, 19].

**Figure 2:**
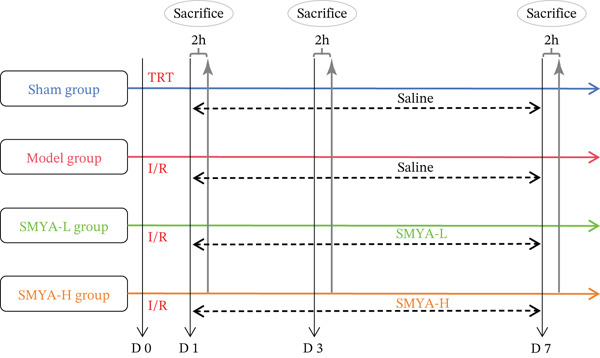
Schematic workflow illustration of the study. After 2 weeks of acclimation, except for the sham group, the left anterior descending (LAD) coronary artery ischemia‐reperfusion was performed for other groups; the sham group underwent the same operation without ligation. Plasma and heart tissue samples were collected at 2 h after the last dose (I/R: ligated left anterior descending coronary artery ischemia‐reperfusion, TRT: thoracotomy, SMYA‐L: low‐dose Si‐Miao‐Yong‐An decoction, SMYA‐H: high‐dose Si‐Miao‐Yong‐An decoction, D: day, h: hour).

Based on the “Pharmacological Experimental Methodology,” the dosages of SMYA for adults were calculated and converted to doses for rats by adjusting for body surface area. For SMYA, the prescribed human dose (90 mg/day) was converted using a body surface area conversion factor (coefficient 0.018). The rat dose was determined as follows:

*Rat dosage = human dosage (mg⁄day) × 0.018⁄(0.2 kg) = 8.1mg⁄(kg/day)/*. This calculated dose falls well within the documented effective range of SMYA (8–24 g/kg) reported in previous cardiovascular studies using rat models [20, 25]. To establish a dose‐response relationship, we selected 8.1 g/kg as the low dose (SMYA‐L) and 16.2 g/kg (twofold higher) as the high dose (SMYA‐H), consistent with standard pharmacological practice for dose gradient design [28]. The model group got the same volume of water as the SMYA‐L group, whereas the concentration of SMYA‐L was given to the former at 8.1 mg/kg. The SMYA‐L group received 8.1 mg/kg/day administered orally, whereas the SMYA‐H group received 16.2 mg/kg/day administered orally. The sham‐operation group (*n* = 6) received water only and was used as model rats. After 7 days, echocardiography was conducted on each rat following 2 h of fasting. At the end of the experimental timeline, following the final echocardiographic assessment, rats were euthanized by exposure to an overdose of isoflurane (5% in oxygen). Animals were placed in a sealed chamber and maintained at this concentration until the cessation of respiration and heartbeat, which was verified by the absence of a pulse and respiratory movements. Following the confirmation of death, blood samples were collected via cardiac puncture, and heart tissues were immediately harvested. Blood and hearts were collected, with tissues preserved in liquid nitrogen for subsequent analysis. To evaluate the temporal effects of SMYA treatment, a serial sacrifice design was employed. Specifically, at each time point (Day 1, Day 3, and Day 7 postmodeling and treatment), three rats from each group were randomly selected and sacrificed for histological analysis and biochemical assays. Consequently, the sample size (*n*) for specific endpoint analyses decreased over time: *n* = 9 at baseline, reducing to *n* = 6 after the Day 1 sacrifice, *n* = 3 after the Day 3 sacrifice, and the remaining *n* = 3 were used for the Day 7 final endpoint. No animals were excluded due to death or surgical failure; all variations in sample size were preplanned experimental endpoints(Figure 2).

A total of 12 rats were assigned to the control group (*n* = 6, 0.9% saline) and SMYA group (*n* = 6). Based on the equal dosage coefficient calculation for experimental rats, the SMYA group (*n* = 6) received 8.1 mg/kg SMYA, equivalent to four times the clinically effective dose, for 3 days. Twelve hours before the experiment, the rats were made to fast. The serum was drawn through the postorbital venous plexus 2 h following the oral dosage. The serum was centrifuged for 10 min at 4°C and 12,000 rpm after collection. After adding 300 *μ*L of serum to three times the volume of acetonitrile, the mixture was vortexed for 2 min, subjected to ultrasonic treatment for 10 min, and finally centrifuged at 12,000 rpm for 10 min. The supernatants were processed by blow‐drying at 40°C with a N2 blower. Subsequently, 50% acetonitrile (50 *μ*L) was added to the residue, followed by 3 min of vortex mixing, 10 min of ultrasonic treatment, and 10 min of centrifugation at 12,000 rpm to collect supernatants for UPLC‐Q‐TOF/MS analysis.

### 2.2. Identification of Major Chemical Components of SMYA Through UPLC‐Q‐TOF/MS

UPLC‐Q‐TOF/MS was employed to identify the chemical components in SMYA‐containing plasma.

#### 2.2.1. Solution Preparation

Forty milliliters of 80% methanol was combined with 1 g of SMYA and then extracted using ultrasonic waves for 30 min. After that, the mixture was spun in a centrifuge at 12,000 rpm for 10 min at 4°C. We collected the supernatant for further study. After adding methanol, the mixture was vortexed for 10 min to separate the drug serum samples from the blank serum. The next step was to spin the mixture in a centrifuge at 12,000 rpm for 10 min at 4°C. After 4 h of vacuum centrifugation, the supernatant was collected. The next step was to add a 50% methanol‐water solution and whirl the mixture for 1 min. For analysis, the liquid above was transferred. Methanol was used to dissolve the relevant standards. The sample solutions were kept in an airtight container in the fridge at 4°C.

#### 2.2.2. Chromatographic and Mass Spectrometric Methods

All three solutions—SMYA, serum sample, and standard—were run through the same UHPLC‐Q‐Orbitrap HRMS system under the same conditions. Here are the conditions for the chromatographic and mass spectrometric analyses:

The ACQUITY UPLC HSS T3 column (2.1 × 100 mm, 1.8 *μ*m particle size) was used in conjunction with a Vanquish Flex UHPLC system to carry out the separation. The volume of the injection was 10 *μ*L. Part A was a mobile phase of acetonitrile and 0.1% formic acid (FA), and Part B was water and 0.1% FA. The mobile phase was mixed at 0.3 mL/min. After a 0–1.0 min period of 2%–2% A, the gradient elution program went as follows: 1.0–41.0 min of 2%–100% A, 41.0–50.0 min of 100%–100% A, 50.0–50.1 min of 100%–2% A, and 50.1–52 min of 2%–2% A.

Acquiring MS data was made possible by connecting a HESI‐II spray probe to a hybrid quadrupole orbitrap mass spectrometer (Q‐Exactive, United States). You may find the set variables here: 3.7 kV for both the positive and negative ions, 320°C for the capillary, 30 psi for the gas surrounding the sheath, 300°C for the desolvation temperature, and so on. Nitrogen at a pressure of 1.5 mTorr made up the sheath, auxiliary, and impact gasses. We made sure to adjust the full scan parameters to 7000 resolution, 1 × 106 auto gain control target, and 50 ms maximum isolation time before we collected the data in “Full scan/dd‐MS2” mode. Acquiring dd‐MS2 data required the following settings: a resolution of 17,500, an auto gain control target of 1 × 105, a maximum isolation period of 50 ms, a loop count of the Top 10 peaks, an isolation window of *m*/*z* 2, collision energies of 10, 30, and 60 V, and an intensity threshold of 1 × 105.

#### 2.2.3. Data Processing and Compound Identification

Importing raw data, extracting peaks, and deconvolving adducts were all phases in the processing of MS data using Progenesis QI 3.0 (Waters, Massachusetts, United States). The identification was completed by taking into account the following factors: reference material retention time error, parent ion mass error, fragment ion match degree, isotope distribution, peak area, and final identification. These factors were compared with a theoretical database built from public and literature sources, as well as the reference substance database (TCM Pro 2.0).

### 2.3. Candidate SMYA Prescription Target Prediction

When it comes to handling complicated illnesses, Chinese herbal medicine places considerable emphasis on the synergy of many components and objectives [29]. The active components of SMYA were found in Section 2.2, and this study used a thorough in silico approach to identify possible targets of these components. An analysis was conducted to determine the target proteins of SMYA using the TCMSP [30], SwissTarget Prediction [31], and DrugBank [32] databases, which were employed to predict the target proteins of SMYA. The GeneCards database [33] and Online Mendelian Inheritance in Man (OMIM) [34] were used to obtain known therapeutic targets for anti‐MI treatment.

### 2.4. Network Establishment and Topological Analysis

It becomes much easier to catch up on the underlying principles of small‐compound regulation and then take advantage of its efficiency and speed through building a network using high‐throughput methods. The SMYA‐associated targets were mapped to the MI‐associated targets to show shared candidate targets by use of a Venn diagram. After that, for further investigation at a system level to show the in‐depth interplay of compounds and their targets, compound‐target networks have been built in Cytoscape 3.8.0. A network analyzer was used for topological analysis to extract the core hub network with the important components and goals [35].

### 2.5. Modular Analysis and Screening of Core Target Genes via the MCODE (Molecular Complex Detection) Algorithm

To identify core hub genes within the network, this study employed the MCODE algorithm for protein–protein interaction network analysis. The core advantage of this algorithm lies in its foundation in local seed proteins and its use of vertex weighting and local neighborhood density calculations, enabling effective identification of highly interconnected functional modules within the network. This approach aligns well with the characteristics of multicomponent, multitarget, and multipathway synergistic actions of TCM. In contrast, tools like CytoHubba focus more on ranking genes based on single topological indicators—such as degree centrality and betweenness centrality—making it difficult to directly reflect the modular structure of synergistic interactions among genes. Specifically, we used MCODE (Version 1.4.2) [36] to identify tightly connected network components. By setting appropriate parameters—including a node score cutoff of 0.2, degree cutoff of 2, and k‐core value of 2—the algorithm precisely identified biologically meaningful core modules from the complex network formed by the 161 consensus targets in this study. Subsequently, using the CytoNCA plugin, preliminary network topology parameters, including intermediate degree connectivity (BC), closed degree connectivity (CC), and degree connectivity (DC), were analyzed. The calculation equations and definitions of these parameters highlight the topological importance of nodes in the hub network. Genes ranked highest in BC, CC, and DC were selected to construct the core network.

### 2.6. Functional Enrichment Analysis

Tools from GENE DENOVO were used for Gene Ontology (GO) and KEGG analysis [37]. Functional enrichment for GO (including biological processes [BP], molecular functions [MF], and cellular components [CC]) and KEGG pathways were plotted and analyzed. The filtering threshold for the search results was set at *p* < 0.05, with the counts organized in descending order.

### 2.7. Echocardiography

For routine transthoracic echocardiography examination, the Vevo2100 ultrahigh‐resolution ultrasound machine (VisualSonics, Canada) was utilized 1, 3, and 7 days after medication intervention. The investigators performing the echocardiography were blinded to the group allocation. Prior to transthoracic echocardiography, animals were put to sleep with isoflurane—2.5% for induction and 2.0% for maintenance with oxygen. The parasternal long‐axis view was acquired using B‐mode echocardiography. The conventional M‐mode method was used to estimate the left ventricular diameter during both diastole and systole. Also measured were left ventricular characteristics such as ejection fraction (EF) and fractional shortening (FS).

### 2.8. Creatine Kinase and Lactate Dehydrogenase (LDH) Measurements

The rat I/R model was successfully prepared, and then within 24 h, blood biochemical testing was carried out. Using an automatic biochemistry analyzer (Mindray, China), the levels of creatine kinase‐MB (CK‐MB) and LDH in serum were determined using commercial kits from Dirui Bioengineering in China. International units (U/L) were used to express the results.

### 2.9. HE Staining and TUNEL Assay

Before being embedded in paraffin, cardiac tissue was preserved in 4% paraformaldehyde. For HE staining, 5 *μ*m slices were cut. Use of the TUNEL assay allowed for the detection of late‐stage apoptosis. A popular apoptosis stain is terminal deoxynucleotidyl transferase (TdT) dUTP Nick‐End Labeling (TUNEL) [38]. DNA in dying cells fluoresces because TUNEL adds dUTPs to the 3 ^′^ hydroxyl termini of DNA.

### 2.10. Western Blotting

After 30 minutes of ice‐based ultrasound lysis of cardiac tissue, protein lysis buffer was added. The protein content in the supernatants of the tissue lysates was measured using the BCA protein assay kit. The proteins were separated by electrophoresis on polyacrylamide gels containing 8% sodium dodecyl sulfate, and then transferred onto polyvinylidene fluoride (PVDF) membranes. Before incubating with primary antibodies for the night at 4°C, membranes were blocked with 5% defatted milk for 2 h. The antibodies consisted of the following: AKT (1:1,000), phospho‐AKT (1:1,000), phosphatidylinositol‐3 kinase (PI3K) (1:500), phospho‐PI3K (1:500), BAX (1:1,000), B‐cell lymphoma‐2 (Bcl‐2) (1:1,000), full‐length caspase‐3 (1:1,000), and anti‐*β*‐actin (1:2,000). The next step was to incubate the membranes for 60 min with a secondary antibody that was 1:4000 and labeled with horseradish peroxidase. For exposure imaging, we employed an automated gel imaging equipment, and for band visualization, we used ECL luminous reagent. Image Lab software was used for protein band analysis. The investigators performing the Western blot analysis were blinded to the group allocation. We conducted a minimum of three separate WB experiments.

### 2.11. Molecular Docking Verification

By looking at the four compounds with the greatest degree values in the PPI network for molecular docking, this study was able to select five core molecules from SMYA. To handle the SMYA active compound Mol2 files, we imported them into AutoDockTools 1.5.7 from TCMSP and saved them in pdbqt format. We used the PDB to get the three‐dimensional structures of AKT, BCL‐2, and BCL‐2 L1. After modifying the target protein in Pymol to remove organic and water molecules, it was loaded into AutoDockTools 1.5.7 to designate a receptor and saved as a pdbqt file [39]. Molecular docking was carried out using AutoDockTools 1.5.7, and the outcomes were displayed using Pymol and LigPlus [40].

### 2.12. Statistical Analysis

Data are expressed as mean ± standard error. To compare two groups, a two‐tailed Student′s *t*‐test was utilized, whereas for comparisons involving multiple groups, one‐way analysis of variance (ANOVA) followed by the Newman–Keuls post hoc test was employed. Additionally, a two‐way ANOVA was employed in this study to evaluate the effects of different treatment groups (sham operation group, model group, SMYA‐L group, and SMYA‐H group) on cardiac EF and short axis FS at various time points (1, 3, and 7 days after I/R). Statistical analyses were performed using GraphPad Prism 10.0. *p* < 0.05 was considered statistically significance.

## 3. Results

### 3.1. Qualitative Analysis on Potential Active Components of SMYA

**Figure 3:**
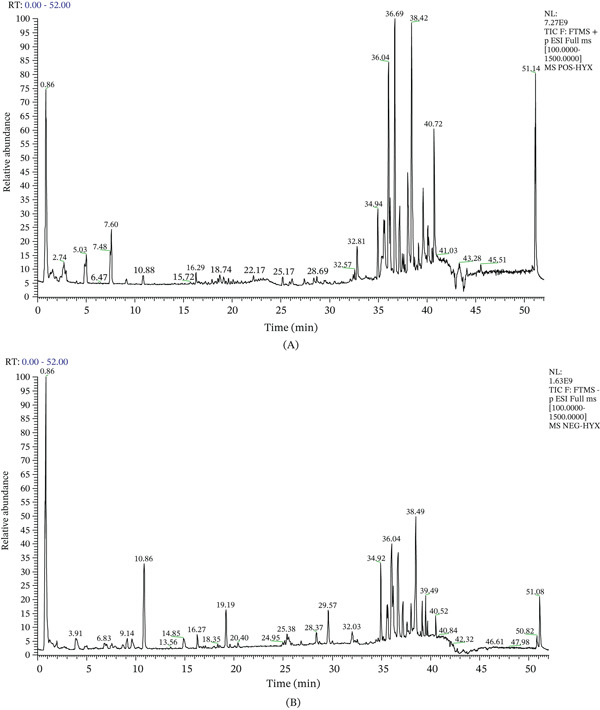
Identification of active compounds in SMYA using UPLC‐Q‐TOF/MS. (A) SMYA in ESI+ mode. (B) SMYA in ESI− mode.

UPLC‐Q‐TOF/MS analysis of plasma samples from SMYA‐treated rats led to the identification of 25 components, based on the precise mass‐to‐charge ratios (*m*/*z*) of fragments and precursors ions. The representative total ion current chromatograms (TIC) acquired in both positive (ESI−) and negative ionization (ESI−) modes are presented in Figure 3A,B. The analysis revealed that flavonoids, phenolic acids, and terpenoids constituted the major classes of compounds in SMYA. Detailed characterizations for all 25 components are provided in Supporting Information 1, and these compounds were subsequently regarded as candidate bioactive constituents for further investigation.

### 3.2. Compound‐Target Network Establishment

**Figure 4:**
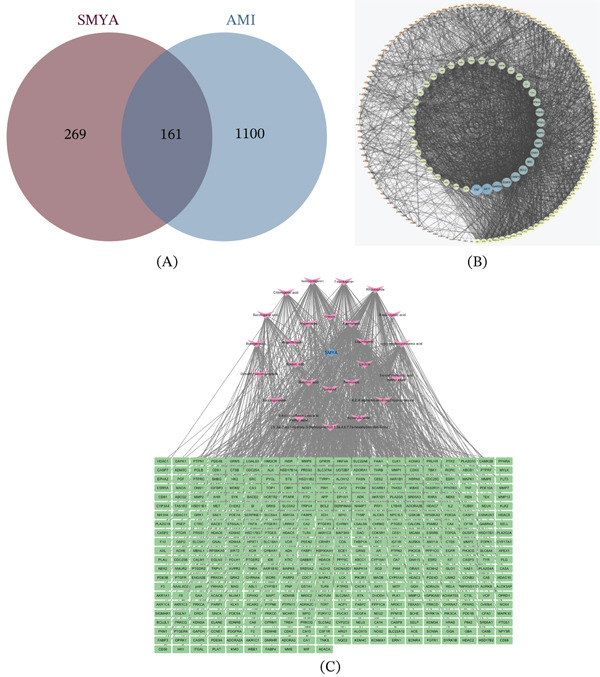
Targets related to AMI and active ingredient‐targets of SMYA. (A) Venn diagram of AMI targets and SMYA targets. (B) The core and noncore target networks. (C) Herb‐ingredient‐targets gene network. The circles above represent the herbs and active compounds of SMYA. The green quadrangles in the middle represent the targets.

A total of 433 targets corresponding to the 25 identified components were retrieved using SwissTargetPrediction (Supporting Information 2). These targets were compiled and visualized as a herbal compound‐target‐disease network using Cytoscape 3.7.2, resulting in a network comprising 456 nodes and 967 edges. After eliminating redundant entries and selecting targets with above‐average scores, 1261 potential MI targets were identified from the Genecards, OMIM, and DisGeNET databases (Supporting Information 3). A Venn diagram was constructed to identify overlapping targets between SMYA and MI (Figure 4A). This analysis yielded 161 common targets, which were identified as potential therapeutic targets of SMYA for MI treatment. The network topology was analyzed to distinguish core from noncore targets, as visualized in the target interaction network (Figure 4B). Additionally, the comprehensive herb‐ingredient‐target gene network illustrates the multiscale therapeutic relationships, with the upper circles representing SMYA herbal constituents and active compounds, and the middle green quadrangles denoting the potential protein targets (Figure 4C, Supporting Information 4).

### 3.3. Core Target Gene Analysis

**Figure 5:**
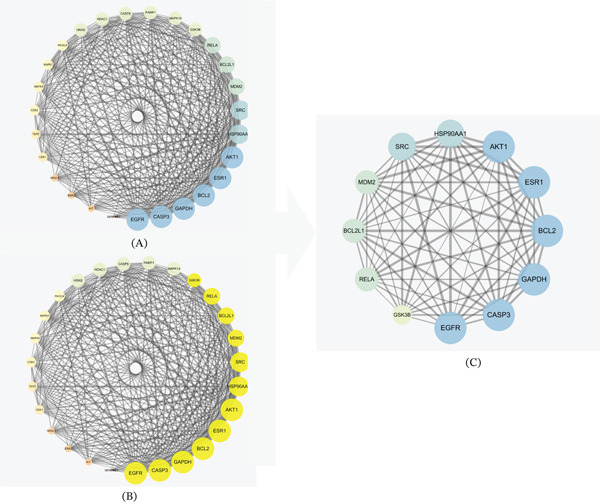
Screening the core PPI network of SMYA′s targets for the treatment of AMI. The highest‐scoring cluster module was identified through MCODE plugin analysis, with an MCODE score of 23.000. The hub module clustering the 27 key targets was determined to be crucial for the treatment of AMI with SMYA. Using the CytoNCA plugin, the Top 12 highly connected nodes were selected as the core network.

Module analysis using the MCODE plugin identified a key clustering module, which achieved the highest score of 23.000. This module contained 27 key targets, crucial for SMYA treatment of AMI. The Top 12 highly connected nodes were selected as the core network through the CytoNCA plugin, including AKT1, ESR1, BCL2, GAPDH, CASP3, EGFR, SRC, HSP90AA1, RELA, BCL2L1, MDM2, and GSK3*β*. Following data screening, functional analysis was conducted on these 12 key target genes to further understand their biological behavior (Figure 5, Table 2).

**Table 2:** Information of 12 core targets.

Swiss prot	Name	Description	Degree	Betweenness centrality	Closeness centrality
P31749	AKT1	Serine/threonine‐protein kinase AKT	26.0	8.483398	1.0
P03372	ESR1	Estrogen receptor alpha	26.0	8.483398	1.0
P10415	BCL2	Apoptosis regulator Bcl‐2	26.0	8.483398	1.0
P04406	GAPDH	Glyceraldehyde‐3‐phosphate dehydrogenase	26.0	8.483398	1.0
P42574	CASP3	Caspase‐3	26.0	8.483398	1.0
P00533	EGFR	Epidermal growth factor receptor	26.0	8.483398	1.0
P12931	SRC	Proto‐oncogene tyrosine‐protein kinase Src	25.0	7.756707	0.962963
P07900	HSP90AA1	Heat shock protein HSP 90‐alpha	25.0	4.50324	0.962963
Q04206	RELA	Nuclear factor NF‐kappa‐B p65 subunit	24.0	3.2136796	0.9285714
Q07817	BCL2L1	Bcl‐2‐like protein 1	24.0	3.2015	0.9285714
Q00987	MDM2	p53‐binding protein Mdm‐2	24.0	4.158363	0.9285714
P49841	GSK3B	Glycogen synthase kinase‐3 beta	23.0	2.8896832	0.8965517

### 3.4. Functional Enrichment

**Figure 6:**
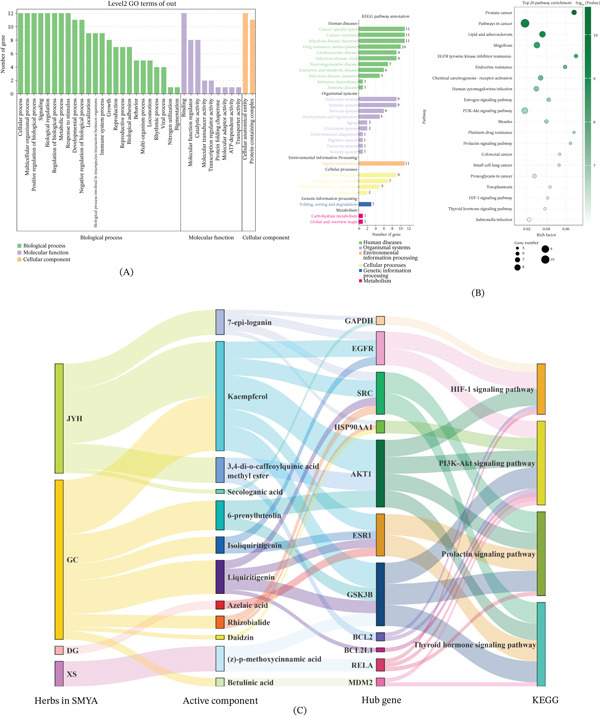
GO and KEGG enrichment analysis of targets. (A) GO enrichment analysis. (B) KEGG pathway analysis. (C) Sankey diagram for KEGG signaling pathway analysis. A key interaction network of traditional Chinese medicine compounds targets pathways has been constructed. A Sankey diagram was constructed using hub genes and relatively active compounds corresponding to four core‐signaling pathways.

GO analysis identified 2623 items, with 1953 showing statistical significance. BP analysis included 2161 items, 1674 of which were statistically significant. MF enrichment analysis revealed 259 items, with 168 being statistically significant. CC analysis indicated 203 items, 111 of which were statistically significant. These findings suggested that GO primarily operates through BP, MF, and CC (Figure 6A). KEGG analysis identified 167 pathways linked to the 12 core targets, with 124 being statistically significant. The Top 20 signaling pathways are illustrated in a bubble chart (Figure 6B). Excluding disease‐related pathways, four key pathways remained significant: PI3K‐Akt, prolactin, HIF‐1, and thyroid hormone. These pathways are predicted to be critical for treating AMI with SMYA. A detailed herb‐compound–target‐pathway interaction network was constructed to elucidate the interactions between SMYA and AMI. Furthermore, Sankey diagrams highlight the hub genes corresponding to the four core signaling pathways and their associated active compounds (Figure 6C).

### 3.5. SMYA Improved Heart Function in I/R Rats

**Figure 7:**
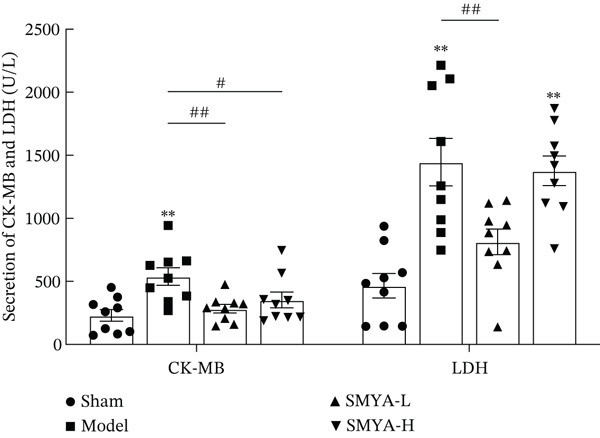
The therapeutic effects of SMYA on I/R rat′s myocardial enzyme. (Sham: *n* = 9; model: *n* = 9; SMYA‐L: low‐dose Si‐Miao‐Yong‐An decoction group, *n* = 9; SMYA‐H: high‐dose Si‐Miao‐Yong‐An decoction group, *n* = 9; h: hour, ##*p* < 0.01, when versus sham, #*p* < 0.05, when versus sham, ∗∗*p* < 0.01, when versus model, ∗*p* < 0.05, when versus model, all data were shown as the means ± SD, and statistical analysis was performed by one‐way analysis of variance [ANOVA] tests).

AMI induced significant increases in circulating levels of CK‐MB and LDH [41]. Four hours postmodeling, saline was administered by gavage to both the sham and model groups, whereas the SMYA‐L and SMYA‐H groups received their respective treatments by gavage. Serum CK‐MB and LDH levels were measured 6 h postsurgery to evaluate the short‐term efficacy of these treatments on the I/R rat model (Figure 7). The model group exhibited significantly elevated levels of CK‐MB and LDH, confirming the successful establishment of the I/R rat model induction. Compared with the model group, both the SMYA‐L and SMYA‐H groups exhibited a significant reduction in CK‐MB levels, indicating that SMYA‐L and SMYA‐H effectively mitigate myocardial injury during the early phase of I/R.

**Figure 8:**
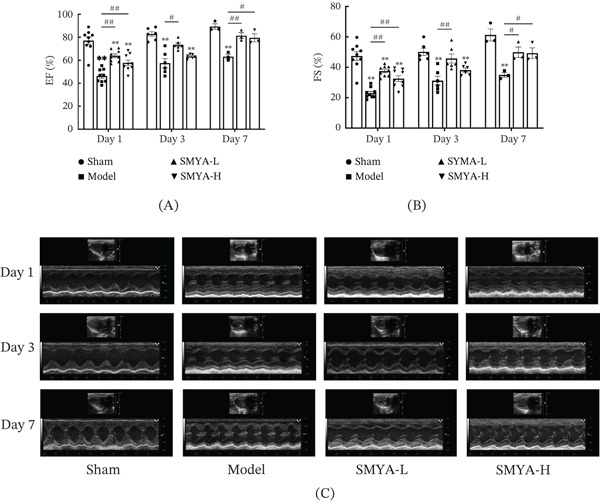
The therapeutic effects of SMYA on I/R rat′s cardiac function. (A) Left ventricular ejection fraction (LVEF) (%), (B) left ventricular fractional shortening (LVFS) (%), (C) representative M‐mode ultrasonic image in short‐axis view. (SMYA‐L: low‐dose Si‐Miao‐Yong‐An decoction group, SMYA‐H: high‐dose Si‐Miao‐Yong‐An decoction group; the sample sizes corresponding to each time point were as follows: *n* = 9 per group on Day 1, *n* = 6 per group on Day 3, and *n* = 3 per group on Day 7; h: hour, ##*p* < 0.01, when versus sham, #*p* < 0.05, when versus sham, ∗∗*p* < 0.01, when versus model, ∗*p* < 0.05, when versus model, all data were shown as the means ± SD, and statistical analysis was performed by two‐way analysis of variance (ANOVA) tests).

Echocardiographic assessment conducted on Days 1, 3, and 7 postsurgery revealed a marked decline in EF and FS in the model group, indicating severe impairment of cardiac function (Figure 8A–C). In contrast, SMYA treatment reversed these reductions in EF and FS, with cardiac function gradually improving over time. SMYA exerts a beneficial impact on cardiac function and helps combat cardiac remodeling following I/R (Figure 8A,B). Therefore, early administration of SMYA after I/R effectively enhances cardiac function and mitigates negative cardiac remodeling induced by I/R.

**Figure 9:**
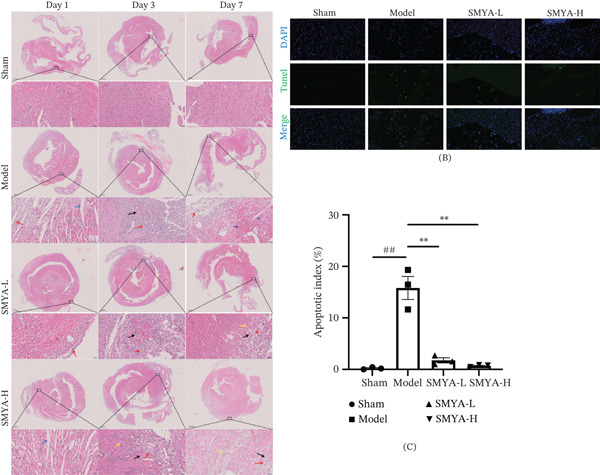
Representative images of (A) H&E staining (magnification × 200) and (B) sections of heart tissue were immunostained with DAPI (blue) and Tunel (green) (Scale bar, 500 *μ*m). (Sham: *n* = 3; model: *n* = 3; SMYA‐L: low‐dose Si‐Miao‐Yong‐An decoction group, *n* = 3; SMYA‐H: high‐dose Si‐Miao‐Yong‐An decoction group, *n* = 3; D: day; Red arrow: Inflammatory cell infiltration can be seen in the tissue; Purple arrow: The nucleus of the myocardial cell is pyknotic, and the cytoplasm is stained with eosin; Black arrow: Visible angiogenesis; Yellow arrow: Hypertrophy of myocardial cells; Blue arrow: Dilation of myocardial interstitium).

To evaluate structural and morphological changes, TUNEL and H&E staining were performed (Figure 9). The model group exhibited significant morphological damage and apoptotic activity, whereas the sham group showed no significant myofiber injury or apoptosis. Treatment with SMYA‐L and SMYA‐H significantly improved myocardial cell damage, promoted capillary neovascularization, and reduced apoptosis. These results indicate that SMYA can effectively enhance cardiac function through multiple pathways in the early stages of I/R.

### 3.6. SMYA Modulates the Phosphorylation of the PI3K‐AKT Pathway During I/R

**Figure 10:**
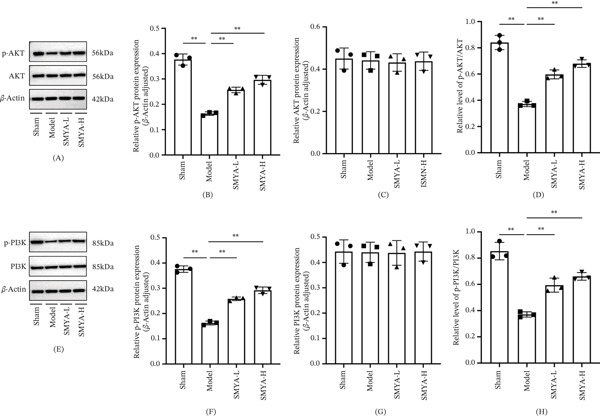
SMYA treatment activated the PI3K/AKT pathway in vivo. (A and E) The total (PI3K and AKT) and phosphorylated (p‐PI3K and p‐AKT) PI3K/AKT pathway proteins in heart tissue were detected by WB. (B) Statistical chart of relative expression level of p‐AKT protein (β‐Actin adjusted). (C) Statistical chart of relative expression level of AKT protein (β‐Actin adjusted). (D) Statistical chart of relative expression level of p‐AKT/AKT protein. (F) Statistical chart of relative expression level of p‐PI3K protein (β‐Actin adjusted). (G) Statistical chart of relative expression level of PI3K protein (β‐Actin adjusted). (H) Statistical chart of relative expression level of p‐PI3K/PI3K protein. All data are shown as mean ± SD (∗*p* < 0.05, ∗∗*p* < 0.01) and statistical analysis was performed by one‐way analysis of variance (ANOVA) tests. (Sham: *n* = 3; model: *n* = 3; SMYA‐L: low‐dose Si‐Miao‐Yong‐An decoction group, *n* = 3; SMYA‐H: high‐dose Si‐Miao‐Yong‐An decoction group, *n* = 3).

PI3K/AKT pathway plays a crucial role in regulating cell growth, survival, and all types of apoptotic processes [42–44]. As shown in Figure 10, the levels of phosphorylated phosphatidylinositol‐3 kinase (p‐PI3K) and AKT (p‐AKT) were significantly reduced in the I/R model rats. In contrast, treatment with both SMYA‐L and SMYA‐H notably enhanced the phosphorylation of PI3K and AKT. The PI3K‐AKT pathway has been implicated in the regulation of cardiovascular functions and the induction of cardioprotection [45]. Enhanced activity of this pathway has been associated with marked cardioprotective effects [46].

In the I/R rat model, inhibition of the PI3K‐AKT pathway was observed, as evidenced by decreased myocardial levels of the active forms of p‐PI3K and p‐AKT. This inhibition was reversed by SMYA treatment, which increased the phosphorylation of PI3K and AKT (Figure 10A–H).

Furthermore, SMYA exerted cardioprotective effects by modulating the expressions of BCL‐2 and BAX, which are target genes of the PI3K‐AKT pathway in regulating cellular apoptosis. Mechanistically, SMYA upregulated BCL‐2 expression and downregulated BAX expression through the PI3K‐AKT pathway, thereby contributing to myocardial protection.

**Figure 11:**
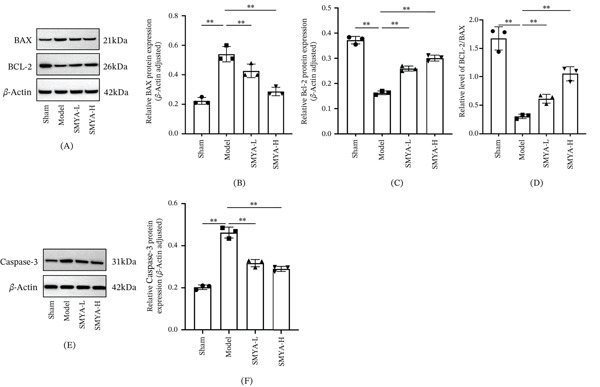
SMYA treatment activated the PI3K/AKT in vivo, and affect cell apoptosis. (A) The total BAX and BCL‐2 proteins in heart tissue were detected by WB. (B) Statistical chart of relative expression level of BAX protein (*β*‐actin adjusted). (C) Statistical chart of relative expression level of BCL‐2 protein (*β*‐actin adjusted). (D) Statistical chart of relative expression level of BCL‐2/BAX protein. (E) Effects of SMYA on the heart tissue of the I/R‐induced rat by determination of antiapoptosis markers (full‐length Caspase‐3). (F) Statistical chart of relative expression level of Caspase‐3 protein (*β*‐actin adjusted). All data are shown as mean ± SD (^*^ *p* < 0.05, ^**^ *p* < 0.01) and statistical analysis was performed by one‐way analysis of variance (ANOVA) tests. (Sham: *n*=3; model: *n*=3; SMYA‐L: low‐dose Si‐Miao‐Yong‐An decoction group, *n*=3; SMYA‐H: high‐dose Si‐Miao‐Yong‐An decoction group, *n*=3).

**Figure 12:**
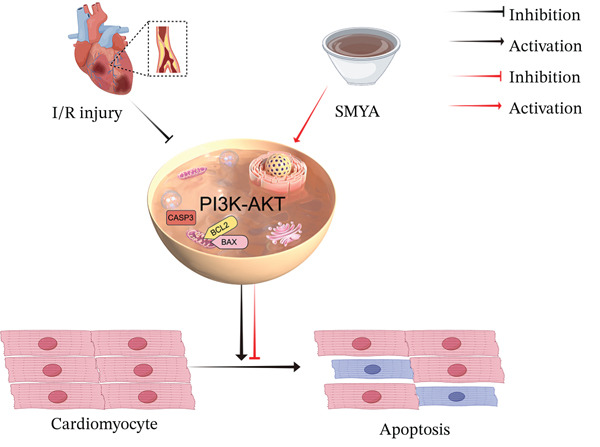
Mechanism of SMYA in treating acute myocardial infarction—regulating myocardial cell apoptosis via PI3K/AKT signaling pathway.

Consistently, WB analysis showed that compared with the model group, the levels of BCL‐2 were significantly down regulated, whereas BAX and caspase‐3 were markedly upregulated in the model group (Figure 11A–F). In contrast, there was a marked increase in protein expression of BCL‐2 with significant reduction in both BAX and caspase‐3 protein levels in the SMYA‐L and SMYA‐H treatment group. Furthermore, SMYA had a dose‐dependent effect, with high doses proving to be the most efficacious. Collectively, these results suggest that SMYA exerts cardioprotection against I/R injury by activating the PI3K‐AKT pathway and regulating the expression of apoptosis‐related proteins, including BAX, BCL‐2, and caspase‐3 (Figure 12).

### 3.7. Predicting Active Compounds of SMYA in the PI3K‐AKT‐BCL‐2 Pathway

**Figure 13:**
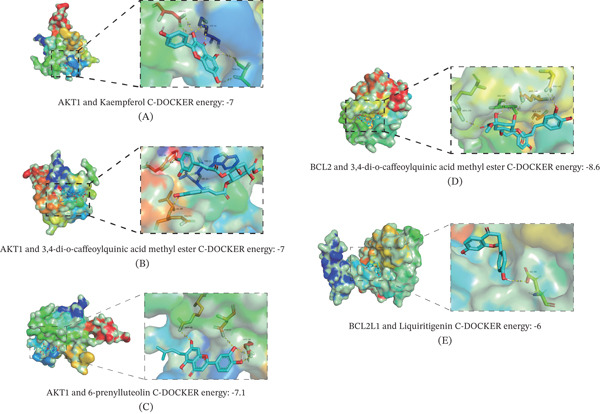
Molecular docking results of main chemical components with (A) kaempterol‐AKT. (B) 3,4‐di‐o‐caffeoylquinic acid methyl ester ‐AKT. (C) 6‐prenylluteolin‐AKT. (D) 3,4‐di‐o‐caffeoylquinic acid methyl ester ‐BCL2. (E) Liquiritigenin ‐BCL2L1.

Based on PPI network analysis, four compounds—kaempferol, 3,4‐di‐o‐caffeoylquinic acid methyl ester, 6‐prenylluteolin, and liquiritigenin—were identified as exhibiting the highest degrees of interaction and were therefore selected for molecular docking simulations with AKT and BCL‐2 proteins. According to established criteria, the lower the binding energy is between a ligand and receptor, the more stable the binding conformation is [47]. The molecular docking results demonstrated favorable binding activities between these components and the target proteins. Specifically kaempferol, 3,4‐di‐o‐caffeoylquinic acid methyl ester, 6‐prenylluteolin, and liquiritigenin showed stable binding to AKT, whereas 3,4‐di‐O‐caffeoylquinic acid methyl ester and liquiritigenin effectively bound to BCL‐2 (Figure 13). The specific binding energies for each compound‐target pair are summarized in Supporting Information 5 and Table 3. These computational results provide structural insights into the potential of these core SMYA components to regulate the PI3K‐AKT pathway and modulate apoptosis‐related processes.

**Table 3:** The molecular docking results.

Gene ID	PBD ID	Compound	C‐DOCKER energy
AKT1	2uzr	Kaempferol	−7
AKT1	2uzr	3,4‐di‐o‐caffeoylquinic acid methyl ester	−7
AKT1	2uzr	6‐prenylluteolin	−7.1
BCL2	6qgg	3,4‐di‐o‐caffeoylquinic acid methyl ester	−8.6
BCL2L1	5c3g	Liquiritigenin	−6

## 4. Discussion

MI is associated with high hospitalization and mortality rates and poor prognosis, with a tendency to progress toward heart failure—a huge burden on patients and society [48, 49]. Although evidence‐based therapies have contributed to a decline in the mortality rate of patients with acute MI, rehospitalization rates and cardiac risks remain high [50]. Myocardial I/R injury is a critical clinical condition that occurs as a result of disruption followed by restoration of blood flow to the heart. Paradoxically, reperfusion causes aggravated cardiac injury through a complex pathophysiological process including oxidative stress, inflammation, apoptosis, and adverse cardiac remodeling [51]. This process significantly contributes to the morbidity and mortality associated with AMI and other IHDs [52]. Consequently, there is an urgent need for effective therapeutic strategies that can mitigate I/R injury and improve clinical outcomes [53]. Although evidence‐based therapies have reduced mortality in MI, rehospitalization rates and cardiac risk remain high, posing a substantial burden on patients [54].

There has been an obvious benefit of TCM therapy on cardiovascular diseases. This might significantly improve the outcomes in the treatment period, decrease readmission rates, and enhance life quality with resultant prolongation of life expectancy [55, 56]. Following MI, key pathological events include cardiomyocyte death and dysfunction, as well as a reactive angiogenic response originating from the infarct border zone and extending into the necrotic core [57, 58]. Recently, network pharmacology has been increasingly applied to investigate the underlying complex mechanism of the therapeutic effect of TCM on the treatment of multivariate diseases. In this study, we applied a network pharmacology‐based approach to systematically explore the anti‐I/R injury effects of SMYA decoction [59]. Using UPLC‐Q‐TOF/MS, we identified 25 major active constituents in SMYA, primarily flavonoids, phenolic acids, and terpenoids. Through network analysis and molecular docking, four components—kaempferol, 3,4‐di‐O‐caffeoylquinic acid methyl ester, 6‐prenylluteolin, and liquiritigenin—were prioritized as key candidates, demonstrating high binding affinity to AKT and BCL‐2, and suggesting their potential role in modulating the PI3K‐AKT pathway.

In the present study, we have shown that SMYA markedly improves heart functional deterioration, possibly due to the activation of the PI3K‐AKT pathway. Our findings indicate that SMYA significantly lowers the serum levels of CK‐MB and LDH, biomarkers of MI, thereby suggesting the role of SMYA in the limitation of myocardial cell injury within the early phase of I/R. Echocardiographic examination showed that SMYA elevated LVEF and FS in response to I/R injury, suggesting that SMYA significantly improved cardiac function and limited adverse remodeling. Furthermore, its effects were characterized by time dependence. Such a conclusion was further confirmed by the histopathological examination. Angiogenesis was increased, whereas apoptosis was decreased in the prolonged SMYA‐treated rats. The myocardial structure was improved [23].

The cardioprotective effects of SMYA are likely mediated through modulation of the PI3K‐AKT pathway. WB analysis revealed that SMYA increased the phosphorylation levels of PI3K and AKT, which are key regulators of cell survival and apoptosis reduction. Additionally, the upregulation of BCL‐2 and downregulation of BAX and caspase‐3 in SMYA‐treated rats further supports the involvement of the PI3K‐AKT pathway in antiapoptotic mechanisms. These molecular changes suggest that SMYA helps preserve myocardial tissue and function by enhancing cell survival pathways and reducing oxidative stress.

However, several important limitations of this study must be acknowledged. First, although network pharmacology provides a powerful tool for hypothesizing drug–target–disease interactions, its predictive nature entails inherent uncertainties. The approach relies on existing databases that are often incomplete or biased toward well‐studied targets, and the computational models used to identify “key targets” require experimental confirmation. Second, although molecular docking suggested binding between SMYA compounds and AKT/BCL‐2, these results remain theoretical without further validation through techniques such as surface plasmon resonance or competitive binding assays. Moreover, the translational relevance of these findings is constrained by the lack of dose‐ranging experimental designs and the unknown contribution of individual compounds to the overall efficacy of SMYA. The multicomponent nature of SMYA poses both an opportunity and a challenge: although it may enable synergistic effects across multiple targets, it also complicates the precise attribution of therapeutic effects to specific chemical constituents. Beyond the PI3K‐AKT pathway, other signaling axes—such as Wnt/*β*‐catenin and HIF‐1*α*—have been implicated in I/R injury and may also be influenced by SMYA [45]. In particular, mitochondrial dysfunction and energy metabolism dysregulation are central to I/R pathology [5, 60, 61], yet the extent to which SMYA modulates these processes remains unclear and warrants further investigation. This study provides evidence that SMYA alleviates myocardial I/R injury, at least in part, through activation of the PI3K‐AKT pathway and inhibition of apoptosis. However, future studies should aim to deconstruct the formula, evaluate the pharmacokinetics and pharmacodynamics of its individual components, and assess potential drug–herb interactions. Long‐term preclinical studies and rigorously designed clinical trials will be essential to determine the safety, efficacy, and therapeutic potential of SMYA in human populations.

## 5. Conclusion

In conclusion, this study integrated analytical chemistry research with network pharmacology and experimental validation to systematically investigate the potential mechanism of SMYA in treating AMI. Using UPLC‐Q‐TOF/MS, we identified 25 major components in SMYA, and subsequent network pharmacology analysis revealed multitarget mechanisms underlying its cardioprotective effects. Experimental results demonstrated that SMYA attenuates myocardial apoptosis in a dose‐ and time‐dependent manner, primarily through activation of the PI3K‐AKT signaling pathway and its downstream effector BCL‐2. These findings not only clarify part of the molecular basis for SMYA′s cardioprotective properties but also provide a valuable foundation for its further clinical translation in the management of ischemic heart disease.

**Nomenclature**
ACS: acute coronary syndromes
AKT: also known as protein kinase B (PKB)
AMI: acute myocardial infarction
ANOVA: analysis of variance
Bcl‐2: B‐cell lymphoma‐2
CK‐MB: creatine kinase‐MB
DDA: data‐dependent acquisition
EF: ejection fraction
FS: fractional shortening
GO: gene ontology
I/R: cardiac ischemia/reperfusion
KEGG: Kyoto Encyclopedia of Genes and Genomes
LDH: lactate dehydrogenase
MI: myocardial infarction
NSTEMI: non‐ST‐segment elevation myocardial infarction
OD: optical density
PI3K: phosphatidylinositol‐3 kinase
ROS: reactive oxygen species
SEM: standard error of the mean
SMYA: Simiao Yong′an decoction
STEMI: ST‐segment elevation myocardial infarction
TCM: traditional Chinese medicine
TIC: total ion current chromatograms
UPLC‐Q‐TOF/MS: ultra‐performance liquid chromatography‐quadrupole‐time‐of‐flight mass spectrometry

## Author Contributions

**Huanjie Fu:** visualization, writing – review & editing, formal analysis, writing – original draft. **Eryue Liu:** visualization, writing – review & editing, formal analysis, writing – original draft. **Hao Yu:** visualization, writing – review & editing. **Yisheng Zhao:** investigation. **Yongkang Gan:** investigation. **Jinhong Chen:** data curation, writing – review & editing. **Zhichao Liu:** visualization, writing – review & editing. Huanjie Fu and Eryue Liu have contributed to the work equally and should be regarded as co‐first authors.

## Funding

This study was supported by the Science & Technology Development Fund of Tianjin Education Commission for Higher Education (2023KJ169); Second Affiliated Hospital of Tianjin University of Traditional Chinese Medicine (YC‐YB202301); Medical and Health Science and Technology Development Project of Shandong Province (10.13039/501100019446, 202303011361).

## Ethics Statement

All male Sprague–Dawley (SD) rats were obtained from Henan Skobes Biotechnology Co. Ltd. (Henan, China; Production License No. SCXK (YU) 2020‐0005), and were housed and used at the facility authorized under the use permit No. SYXK (JINBIN) 2023‐0005. Our experimental protocols were approved by the Ethics Committee for the use of experimental animals at the Tianjin Jinke Bona Biotechnology Co. LTD. (Approval No.: GENINK20230060; Date: 07/12/2023).

## Conflicts of Interest

The authors declare no conflicts of interest.

## Supporting Information

Additional supporting information can be found online in the Supporting Information section.

## Supporting information


**Supporting Information 1** Flavonoids, phenolic acids, and terpenoids were shown to be the main constituents of SMYA. Detailed characterizations of 25 components are presented in Supporting Information 1, and they served as candidate effective components.

**Supporting Information 2** There were 433 targets of 25 components detected based on SwissTargetPrediction, collected and visualized as herbal compound target disease with Cytoscape 3.7.2.

**Supporting Information 3** A total of 1261 potential targets of MI in Genecards, OMIM, and DisGeNET were acquired after removing redundant entries and filtering targets with scores higher than average.

**Supporting Information 4** A total of 161 consensus targets were identified to be possible therapeutic targets of SMYA for the treatment of MI, which were employed to construct a compound‐target network.

**Supporting Information 5** The molecular docking results are displayed in Supporting Information 5.

## Data Availability

The data that support the findings of this study are available from the corresponding authors upon reasonable request.
